# Correlation among 16 biological factors [p53, p21^waf1^, MIB-1 (Ki-67), p16^INK4A^, cyclin D1, E-cadherin, Bcl-2, TNF-α, NF-κB, TGF-β, MMP-7, COX-2, EGFR, HER2/neu, ER, and HIF-1α] and clinical outcomes following curative chemoradiation therapy in 10 patients with esophageal squamous cell carcinoma

**DOI:** 10.3892/ol.2013.1130

**Published:** 2013-01-11

**Authors:** SHINO SHIBATA-KOBAYASHI, HIDEOMI YAMASHITA, KAE OKUMA, KENSHIRO SHIRAISHI, HIROSHI IGAKI, KUNI OHTOMO, KEIICHI NAKAGAWA

**Affiliations:** Department of Radiology, The University of Tokyo Hospital, Tokyo, Japan

**Keywords:** esophageal cancer, imunohistochemistry, squamous cell carcinoma, prognostic factors, biological markers

## Abstract

The expression levels of 16 proteins were analyzed to identify prognostic correlations in esophageal squamous cell carcinoma (ESCC) treated with concurrent chemoradiation therapy (CCRT). The immunohistochemical expression levels of p53, p21^waf1^, molecular immunology borstel-1 (MIB-1, Ki-67), p16^INK4A^, cyclin D1, E-cadherin, Bcl-2, tumor necrosis factor (TNF)-α, nuclear factor (NF)-κB, transforming growth factor (TGF)-β, matrix metalloproteinase (MMP)-7, cyclooxygenase (COX)-2, epidermal growth factor receptor (EGFR), human EGFR type 2 (HER2/neu), estrogen receptor (ER) and hypoxia-inducible factor (HIF)-1α were studied in 10 cases of ESCC treated with CCRT. The patients underwent CCRT between 2000 and 2010. The mean patient age was 68.1 years (range, 46-80 years). The numbers of patients in stages I, II, III and IV of the disease were 2, 2, 3 and 3, respectively. Of the tumors, 8 were positive for p53, 6 for p21^waf1, 7^ for MIB-1 (Ki-67), 7 for p16^INK4A^, 7 for cyclin D1, 8 for E-cadherin, 3 for Bcl-2, 0 for TNF-α, 5 for NF-κB, 7 for TGF-β, 9 for MMP-7, 7 for COX-2, 5 for EGFR, 1 for HER2/neu, 1 for ER and 7 for HIF-1α. The 2-year overall survival rate of patients expressing high levels of MIB-1 was 71% (±17%) compared with 0% (P=0.019) for those expressing low levels. For NF-κB, the rate was 0% for patients with high levels compared with 100% (P<0.018) for those with low levels. The 2-year local control rates of HER2/neu were 0% in patients expressing high levels and 88% (±12%) in patients expressing low levels (P=0.027). The 2-year disease-free survival rates of HER2/neu and ER were 0% for patients expressing high levels compared with 56% (±17%) for those with low levels (P=0.027). There were no significant correlations between the expression levels of the other proteins and clinical outcomes. In the present study, high levels of MIB-1 and low levels of NF-κB, HER2 and ER were shown to be good prognostic factors following definitive CCRT for ESCC.

## Introduction

Various investigators have studied the prognostic factors of esophageal squamous cell carcinoma (ESCC) treated with concurrent chemoradiation therapy (CCRT). In previous studies, it was considered that the expression levels of p53 (1–3), p21^waf1^(4), molecular immunology borstel-1 (MIB-1) (2,5), p16^INK4A^(6), cyclin D1 (2,7,8), E-cadherin (8), Bcl-2 (1), tumor necrosis factor (TNF)-α (9), nuclear factor (NF)-κB (10), transforming growth factor (TGF)-β (11), matrix metalloproteinase (MMP)-7 (12), cyclooxygenase (COX)-2 (13,14), epidermal growth factor receptor (EGFR) (15) and hypoxia-inducible factor (HIF)-1α (16) may be used as prognostic factors for ESCC. However, these studies included patients treated with surgery or neoadjuvant chemoradiation therapy (CRT). In the case of definitive CRT, the prognostic factors may be different from those of surgery or neoadjuvant CRT. Unlike surgical resection alone, which is not reliant on the therapeutic response, and neoadjuvant CRT, which is able to achieve complete responses in the majority of cases by resecting (even for cases without marked responses), in definitive CRT it is important to be able to predict therapeutic responses before starting treatment. Identifying predictive parameters may aid the selection of primary therapies. The present study was conducted using biopsy specimens excised from patients prior to CCRT. The expression levels of 16 proteins were analyzed to identify prognostic correlations in ESCC treated with CCRT.

## Patients and methods

### Patients

A total of 10 patients who received CCRT for ESCC at the University of Tokyo Hospital (Japan) between June 2000 and June 2010 were selected retrospectively. Only 10 patients (5 long-term survivors and 5 who had succumbed to cancer) were selected and examined, since this was a preliminary study to determine which type of immunostaining should be used in the following larger study. ESCC was confirmed histologically in all 10 patients. The patients consisted of 4 cases of good responses and 6 recurrent cases. The patients were staged according to the American Joint Committee for Cancer Staging and End Results Reporting 1997 staging system (17). The initial staging consisted of a patient history and medical examination, routine blood tests, chest X-rays, upper magnifying endoscopy, chest and upper abdomen computerized tomography (CT), barium contrast X-rays and pulmonary function tests. Bone scans and CT or magnetic resonance imaging of the brain were performed only in cases of clinical suspicion of metastases. Patients with technically unresectable cancer, patients who refused to undergo surgery or those considered medically unfit for surgery were eligible for definitive CCRT.

The study was approved by the ethics committee of the University of Tokyo, Tokyo, Japan. Informed consent was obtained from all patients or the patient’s families.

### CRT method

The details of the treatment method have been previously reported (18–20). All patients received extended elective nodal irradiation and were treated with 50–50.4 Gy delivered at 1.8–2 Gy per fraction over 5–5.6 weeks. The clinical target volume (CTV) was defined as the whole thoracic esophagus (from the supra-clavicular fossae to the esophagogastric junction). The CTV comprised the M1a and regional lymph nodes (LNs), including positive LNs. The planning target volume was caluclated by adding margins of 5–10 mm to the respective CTVs. The treatment planning was entirely 3-dimensional. At least 4 fields were used (2 anterior-posterior opposed fields and 2 anterior-posterior oblique opposed fields to remove the spinal cord from the radiation fields) and 1 or 2 beams were added using the field-in-field technique if necessary. Treatment was delivered by linear accelerators with 6–10 MV photons.

All patients received chemotherapy (CTx) concurrently with irradiation. The CTx consisted of 2 cycles of 5-fluorouracil (800 mg/m^2^/day, days 1–4 and 29–32, continuous) combined with nedaplatin (80 mg/m^2^, days 1 and 29, bolus) and standard techniques were used for hydration and alkalization. The CTx began on the first day of irradiation. After CCRT, in the adjuvant setting, an additional 1 or 2 cycles of the same doses of CTx were administered to patients who had sufficient bone marrow function and performance status and did not refuse additional CTx.

### Follow-up

Patients were followed up on a regular basis, with visits at 1 month following treatment, every 3 months thereafter during the first 2 years and every 6 months thereafter. Chest X-rays were performed at every visit, while chest and upper abdomen CT was performed every 6 months or more frequently at the suspicion of tumor progression. When tumor growth was identified by CT and/or PET, this was defined as local recurrence.

### Immunohistochemistry (IHC)

Specimens obtained from biopsies under endoscopy before the treatment were used. Tumor samples were fixed with 10% formaldehyde in phosphate-buffered saline (PBS), embedded in paraffin and 4-*μ*m thick sections were prepared. The sections were deparaffinized and pretreated with various methods, including microwaving, EDTA and heating, that are known to be effective at unmasking reactive sites for antibodies. The characteristics of the 16 primary antibodies used in IHC and their pretreatments are shown in Table I. Subsequently, IHC staining was performed with an automated IHC stainer. The slides were then rinsed briefly in water and counterstained with haematoxylin.

The level of expression was assessed semi-quantitatively using the immunoreactive scoring (IRS) system (21). The IRS score was determined by considering the intensity, graded on a scale of 0–3 (0 = no staining, 1 = weak staining, 2 = moderate staining and 3 = marked staining) and extent (percentage of positive tumor cells) of staining. The extent of staining was graded on a scale of 0–4 (0 = no staining; 1 = 1–10% staining, 2 = 11–50% staining, 3 = 51–80% staining and 4 = 81–100% staining). The IRS score (range, 0–12) was the product obtained by multiplying the intensity and extent of staining.

The IRS scores from 0–12 were interpreted as follows: 0, negative; 1–4, weak; 5–8, moderate; and 9–12, markedly positive.

The low and high cut-off values for the scores of the biochemical markers were established as follows: i) p53, p16^INK4A^, cyclin D1, E-cadherin, TGF-β, MMP-7, COX-2, EGFR and HIF-1α: low, 0–2; high, 3–12; ii) Bcl-2 and HER2/neu: low, 0–8; high, 9–12; iii) MIB-1 (Ki-67): low, 0–3; high, 4–12; iv) p21^waf1^, estrogen receptor (ER) and TNF-α: low, 0–3; high, 4–12; and v) NF-κB: low, 0–11; high, 12.

### Statistical analysis

Statistical analyses of the correlations between the clinical results (alive vs. deceased, with vs. without local recurrence and with vs. without disease) and molecular markers were performed using Fisher’s exact test. The association between each pair of proteins expressed was determined using the Pearson product-moment correlation coefficient.

The Kaplan-Meier product-limit method was used to estimate the probabilities of overall survival (OS), disease-free survival (DFS) and locoregional recurrence-free survival, while the log-rank test was used to estimate any differences. OS was calculated in months from the first day of CCRT to the date of mortality from any cause or to February 2012. Patients who remained alive in February 2012 were censored. P<0.05 was considered to indicate statistically significant differences.

## Results

### Patients

The characteristics of the 10 patients are shown in Table II. The median age was 68.1 years (range, 46–80 years). The sub-sites of the primary tumors included the middle (n=2) or lower (n=8) thoracic portions. The TNM classifications were as follows: T1/T2/T3/T4, 2/3/5/0; N0/N1, 3/7; M0/M1a/M1b, 7/0/3; and stages I/II/III/IV, 2/2/3/3, respectively. The median follow-up for the 5 surviving patients was 76.2 (±24.8) months.

### IHC

Staining evaluations, (negative, weak, moderate or marked for each of the 16 primary antibodies, were performed for the 10 patients. The results may be summarized as follows (with the numbers representing how many of the 10 patients showed expression for the antibody): a) negative: 1 p16^INK4A^, 4 Bcl-2, 4 TNF-α, 1 EGFR, 5 HER2/neu and 4 ER; b) Moderate: 1 p53, 1 p21^waf1^, 3 MIB-1 (Ki-67), 1 p16^INK4A^, 1 cyclin D1, 2 E-cadherin, 4 TGF-β, 4 MMP-7, 6 COX-2, 4 EGFR and 4 HIF-1α; c) Strong: 7 p53, 1 p21^waf1^, 7 MIB-1 (Ki-67), 6 p16^INK4A^, 7 cyclin D1, 7 E-cadherin, 3 TGF-β, 6 MMP-7, 3 COX-2, 4 EGFR and 5 HIF-1α. In addition, correlation analysis revealed the following results with respect to the pairs of expression levels: p53 vs. cyclin D1, r=0.791, P=0.045; EGFR vs. p53, r=0.803, P=0.0034; MIB-1 vs. p21, r=0.752, P=0.0097; HER2 vs. ER, r=0.823, P=0.020; TNF-α vs. p21, r=0.739, P=0.012; TGF-β vs. COX-2, r=0.714, P=0.018; TGFβ vs. HIF-1α, r=0.730, P=0.014; and COX-2 vs. HIF-1α, r=0.794, P=0.0042.

### Clinical outcome

**Figure 1:**
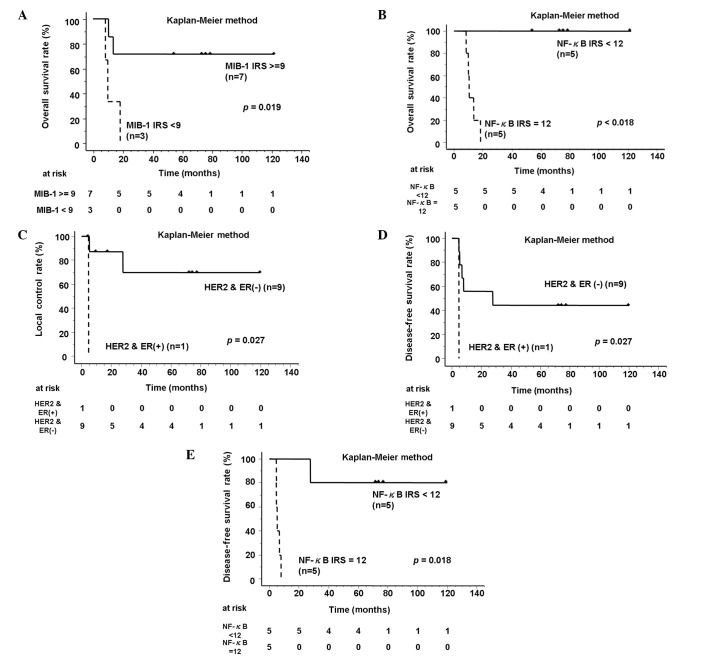
Survival curves for ESCC treated with CCRT. (A) OS curves according to high (IRS ≥9) and low (IRS <9) MIB-1 levels. (B) OS curves according to high (IRS = 12) and low (IRS <12) NF-κB levels. (C) LC curves according to high (HER2/neu IRS ≥3, ER IRS ≥4) and low (HER2/neu IRS = 1 or 2, ER IRS <4) levels of both HER2/neu and the ER. (D) DFS curves according to high and low levels of both HER2/neu and the ER. (E) DFS curves according to both the high and low levels of NF-κB. ESCC, esophageal squamous cell carcinoma; CCRT, concurrent chemoradiation therapy; MIB-1, molecular immunology borstel-1; IRS, immunoreactive score; OS, overall survival; NF-κB; nuclear factor-κB; LC, local control; HER2/neu, human epidermal growth factor receptor type 2; ER, estrogen receptor; DFS, disease-free survival.

Significant 2-year OS rates were observed for patients with high (MIB-1 IRS ≥9) and low (MIB-1 IRS <9) MIB-1 levels (71 vs. 0%, P=0.019; Fig. 1A) and high (NF-κB IRS = 12) and low (NF-κB IRS <12) NF-κB levels (0 vs. 100%, P<0.018; Fig. 1B). The correlations between the OS and expression levels of p53, p21^waf1^, p16^INK4A^, cyclin D1, E-cadherin, Bcl-2, TNF-α, TGF-β, MMP-7, COX-2, EGFR, HER2/neu, ER and HIF-1α were not found to be significant (Table III).

The 2-year local control rates were 0 vs. 88% (±12%, P=0.027; Fig. 1C) for patients with high (HER2/neu IRS ≥3, ER IRS ≥4) and low (HER2/neu IRS ≤2, ER IRS <4) levels of both HER2/neu and ER. The 2-year DFS rates of the same patients were 0 vs. 56% (±17%; P=0.027; Fig. 1D) and 0 vs. 80% (±18%; P=0.018; Fig. 1E) for patients with high and low levels of NF-κB, respectively. No significant correlations were observed between the expression levels of the other 12 proteins and clinical outcomes (Table III).

## Discussion

The clinical outcomes of 10 ESCC patients treated with radical CCRT were correlated with 16 molecular biomarkers. The present retrospective study was performed on patients treated between 2000 and 2010. A number of previous studies were designed for patients treated with radical surgery or preoperative CCRT (1–16). The present study is the first to conduct a comprehensive analysis of 16 proteins simultaneously in patients treated with definitive CCRT-alone. Additionally, the study showed for the first time that 4 biomarkers, including MIB-1, NF-κB, HER2 and ER, are prognostic factors for ESCC treated with CCRT.

In the present study, 10 patients were treated using a standard CCRT regimen. The 2-year OS, local control (LC) and DFS rates were all 50% and these results were comparable to previous reports (18–20). There was little difference in the clinical backgrounds between the 6 living and 4 deceased patients. In the cases of higher MIB-1 expression, the OS was significantly improved following definitive CCRT. In the cases with lower NF-κB expression, the OS and DFS rates were significantly improved following definitive CCRT. In the cases of lower HER2 and ER levels, the LC and DFS rates were significantly improved following definitive CCRT. TNF-α showed negative or extremely weak staining in all cases. There was no correlation between MIB-1 and NF-κB, which were independent prognostic factors. There was a strong correlation between HER2 and ER. All patients with high/low HER2 expression also expressed high/low ER. The results of the HER2 staining were entirely in accordance with those of ER.

MIB-1 monoclonal antibody recognizes proliferating cells in the G1, S, G2 and M phases of the cell cycle (22). The correlation between a high MIB-1 index and poor prognosis is well known in breast cancer (23). However, in the present study, in the cases of higher MIB-1 expression, the OS was significantly improved following definitive CCRT. Ressiot *et al*(5) concluded that the over-expression of MIB-1 was a significant factor for complete endoscopic response following CCRT in 56 patients with esophageal cancer, which is similar to the present results. The correlation between MIB-1 expression and a good response to CCRT is explained by the fact that MIB-1 expression is a marker of cellular proliferation.

NF-κB is a protein complex that controls the transcription of DNA. TNF-α and other transcription factors activate NF-κB. NF-κB activates interleukin (IL)-1 and others and is therefore involved in apoptosis or inflammation. Blocking NF-κB causes tumor cells to stop proliferating, die or become more sensitive to the action of antitumor agents. Thus, NF-κB is the subject of much active research among pharmaceutical companies as a target for anticancer therapy (24). Izzo *et al*(10) reported that activated NF-κB prior to therapy in 80 patients with esophageal cancer was associated with the lack of a complete pathological response, which supports the present result showing that patients with high expression levels of NF-κB had significantly poorer OS and DFS rates than those showing low levels.

HER2 is a member of the EGFR family which is involved in the complex regulation of cell growth, proliferation and survival. HER2 protein has been implicated in the development of cancer. In addition to breast cancer, HER2 overexpression and gene amplification have also been reported in carcinomas of the colon, bladder, ovary, endometrium, lung, head and neck, esophagus and stomach (25). Akamatsu *et al*(26) reported that HER2 immunostaining was suitable for predicting resistance to CRT in 34 patients with ESCC. Additionally, according to Mimura *et al*(27), the survival rate of 66 patients with ESCC showing HER2 gene amplification was significantly worse than those without amplification.

The main function of ER is as a DNA-binding transcription factor regulating gene expression. Estrogen and ER have also been implicated in breast, ovarian, colon, prostate and endometrial cancer. Wang *et al*(28) suggested that ER expression may predict a better outcome for patients with ESCC.

These results concurred with the present results where patients showing high staining for HER2 and ER had significantly poorer LC and DRS rates than those with low staining. Estrogen promotes tumor proliferation through ER and in cells with overexpression of HER2, tumor proliferation is considered to be increased. The present result demonstrating that the prognosis of cases with overexpression of ER or HER2 was worse is therefore understandable.

In the present study, p53, p21^waf1^, p16^INK4A^, cyclin D1, E-cadherin, Bcl-2, TNF-α, TGF-β, MMP-7, COX-2, EGFR and HIF-1α were also investigated. The p53 gene is one of the well-known anti-oncogenes involved in apoptosis and the cell cycle (29–31). Also, the p21 and the p16^INK4A^ proteins are involved in the cell cycle and induce G1 arrest. Cyclin D1 is a member of the cyclin protein family involved in regulating cell cycle progression. Bcl-2 is a member of the anti-apoptotic family (32,33). E-cadherin is important in cell adhesion and loss of E-cadherin function or expression has been implicated in cancer progression and metastasis. TGF-β is important in stopping the proliferation of normal epithelial cells and promoting the invasion of cancer cells. TNF, being an endogenous pyrogen, is able to induce fever, apoptotic cell death, sepsis through IL-1 and 6 production, cachexia and inflammation, as wells as inhibit tumorigenesis and viral replication. The expression of COX-2 is induced by several stimuli, including growth factors, inflammation and cytokines. COX-2 is upregulated in numerous types of cancer (34). EGFR is the cell-surface receptor for epidermal growth factors and is a growth factor involved in the regulation of cell growth, proliferation and differentiation. Proteins of the MMP family are involved in the breakdown of the extracellular matrix in normal physiological processes, such as embryonic development, reproduction and tissue remodeling, as well as in disease processes, such as arthritis and metastasis (35). HIF-1α is a transcription factor that responds to changes in available oxygen in the cellular environment, particularly to decreases in oxygen or hypoxia (36). In the present study, these cancer growth-causing proteins did not appear to be prognostic factors for ESCC treated with CCRT.

However, the present study had a number of limitations. Firstly, the power may be poor since it involved only 10 patients in a retrospective setting. Secondly, the IHC method used was only able to examine protein expression levels and DNA mutation and amplification were not examined as with the polymerase chain reaction method.

We expect to continue these studies, thereby increasing the number of cases in a retrospective setting and confirming the conclusions of the present study.

In the present study, high levels of MIB-1 and NF-κB and low levels of HER2 and ER were good prognostic factors following definitive CCRT for ESCC. There was no significant correlation between the expression levels of the other proteins (p53, p21^waf1^, p16^INK4A^, cyclin D1, E-cadherin, Bcl-2, TNF-α, TGF-β, MMP-7, COX-2, EGFR and HIF-1α) and clinical outcomes.

## Figures and Tables

**Table I:** Immunohistchemistry: characteristics of the primary antibodies.

Protein	Type	Source	Pretreatment	Titer	Incubation	Staining
p53	Mouse MC	DO7 NCL-p53-DO7, Leica	Microwave 1 mM EDTA (pH 8.0)	1:200	30 min, 37°C	Nucleus
p21^waf1^	Mouse MC	SNCL-WAF-1, Novocastra	Heating (121°C, 15 min), citrate buffer (pH6.0)	1:100	Overnight, 4°C	Nucleus
MIB-1	Mouse MC	MIB-1 M7240, DAKO	Microwave, 1 mM EDTA (pH 8.0)	1:25	30 min, 37°C	Nucleus
p16^INK4A^	Mouse MC	Z2117, Zeta	Heating (121°C, 15 min), citrate buffer (pH 6.0)	1:200	Overnight, 4°C	Cytoplasmic and nucleus
cyclin D1	Rabbit MC	SP4 RM-9104-S, Thermo	Microwave, 1 mM EDTA (pH 8.0)	1:250	30 min, 37°C	Cytoplasmic
E-cadherin	Mouse MC	36B5 NCL-E-Cad, Leica	Microwave, 1 mM EDTA (pH 8.0)	1:25	30min, 37°C	Membrane (cytoplasmic)
Bcl-2	Mouse MC	124 M0887, DAKO	Microwave, 1 mM EDTA (pH 8.0)	1:80	30 min, 37°C	Cytoplasmic
TNF-α	Mouse MC	2C8 sc-52250, Santa Cruz	Heating (121°C, 20 min), citrate buffer (pH 6.4)	1:100	Overnight, 4°C	Cytoplasmic
NF-κB	Rabbit PC	sc-7178, Santa Cruz	Heating (121°C, 20 min), Citrate buffer (pH 6.0),	1:1000	Overnight, 4°C	Cytoplasmic
TGF-β1	Rabbit PC	Y241, Yanaihara	None	1:200	Overnight, 4°C	Cytoplasmic
MMP-7	Rabbit PC	AB19135, Chemicon International	None	1:500	Overnight, 4°C	Cytoplasmic
COX-2	Rabbit PC	18515, IBL	None	0.5*μ*g/ml	Overnight, 4°C	Cytoplasmic
EGFR	Mouse MC	K1492, DAKO	Proteinase K (kit)	R-to-U	30 min, RT	Membrane (cytoplasmic)
HER2	Rabbit PC	K5204, DAKO	Water bath (99°C), citrate buffer (pH 6.0)	R-to-U	30 min, RT	Membrane
ER	Mouse MC	790-4325, Roche	Microwave, 0.01 M citrate buffer (pH 6.0)	1:100	60 min, RT	Membrane (cytoplasmic)
HIF-1α	Rabbit PC	07-628, MILLIPORE	None	1:200	Overnight, 4°C	Nucleus

RT, room temperature; R-to-U, ready to use (kit); MC, monoclonal; PC, polyclonal; MIB-1, molecular immunology borstel-1; TNF-α, tumor necrosis factor-α; NF-κB, nuclear factor-κB; TGF-β, transforming growth factor-β; MMP-7, matrix metalloproteinase-7; COX-2; cyclooxygenase-2; EGFR, epidermal growth factor receptor; HER2, human epidermal growth factor receptor type 2; ER, estrogen receptor; HIF-1α, hypoxia-inducible factor-1α; Leica, Mannheim, Germany; Novocastra, Newcastle upon Tyne, United Kingdom; Dako, Carpinteria, CA, USA; Zeta, Sierra Madre, CA, USA; Thermo, Walthem, MA, USA; Santa Cruz Biotechnology, Inc., Santa Cruz, CA, USA; Chemicon International, Temecula, CA, USA; IBL, Minneapolis, MN, USA; Roche, Mannheim, Germany; Millipore, Billerica, MA, USA.

**Table II:** Patient charactaristics.

Patient no.	Age (years)	Gender	Primary site	Stage	cTNM	Hb (units)	BW loss (kg)	Dysphagia score	Tumor length (cm)	Differentiate	Chemotherapy total cylcle	Radiation dose (Gy)	Survival	Local recurrence	Recurrence
1	68	Male	Lt	I	T1N0M0	14.8	0	0	7	M/D	2	50.4	Alive	Without	Without
2	70	Male	Lt	I	T1N0M0	15.9	0	1	7	P/D	3	50	Dead	Without	With
3	46	Male	Lt	IVB	T3N1M1b	10.9	13	3	4	P/D	2	60	Alive	Without	Without
4	66	Male	Lt	IIA	T3N0M0	10.9	0	3	3.5	NA	4	50.4	Dead	Without	With
5	80	Male	Lt	III	T3N1M0	11.3	0	3	5	W/D	4	50.4	Dead	Without	With
6	70	Female	Mt	IVB	T2N1M1b	11.5	0	1	6	W/D	3	50.4	Dead	With	With
7	68	Female	Lt	III	T3N1M0	12.1	0	0	4	M/D	4	50.4	Alive	Without	Without
8	79	Male	Lt	III	T3N1M0	13.5	8	2	7	P/D	2	50.4	Alive	Without	Without
9	69	Male	Mt	IVB	T2N1M1b	11.6	0	1	2	M/D	2	50	Dead	With	With
10	65	Male	Lt	IIB	T2N1M0	14.8	7	2	NA	P/D	4	60	Alive	With	With

Mt, middle thoracic; Lt, lower thoracic; BW, body weight; P/D, poorly differentiated; W/D, well differentiated; M/D, moderately differentiated; NA, not applicable.

**Table III:** Clinical outcome by the expression levels of biomarkers.

IHC	IRS score	No.	2-year OS	SD	P-value log-rank	2 year LC	SD	P-value log-rank	2 year DFS	SD	P-value log-rank
Bcl-2											
High	3–12	3	67	27	0.55	67	27	0.19	67	27	0.85
Low	0–2	7	43	19		83	15		43	19	
Cyclin D1											
High	6–12	7	43	19	0.38	69	19	<0.19	43	19	0.32
Low	0–5	3	67	27		100	0		67	27	
E-cadherin											
High	6–12	8	50	18	0.76	73	17	<0.90	50	18	0.75
Low	0–5	2	50	35		100	0		50	35	
EGFR											
High	6–12	5	60	22	0.57	80	18	0.75	60	22	0.67
Low	0–5	5	40	22		80	18		40	22	
MIB-1											
High	9–12	7	71	17	0.019^a^	71	17	<0.83	71	17	0.90
Low	0–8	3	0	0		100	0		0	0	
p53											
High	6–12	8	50	18	0.76	73	17	<0.98	50	18	0.75
Low	0–5	2	50	35		100	0		50	35	
HER2											
High	3–12	1	0	0	0.22	0	0	0.027^a^	0	0	0.027^a^
Low	0–2	9	56	17		88	12		56	17	
ER											
High	4–12	1	0	0	0.22	0	0	0.027^a^	0	0	0.027^a^
Low	0–3	9	56	17		88	12		56	17	
p16^INK4A^											
High	6–12	7	50	18	0.75	75	15	<0.23	50	17	0.95
Low	0–5	3	50	35		100	0		50	35	
TNF-α											
High	4–12	0	-	-	-	-	-	-	-	-	-
Low	0–3	10	-	-	-	-	-	-	-	-	-
p21^waf1^											
High	4–12	6	50	20	0.70	67	19	<0.16	50	20	0.65
Low	0–3	4	50	25		100	0		50	25	
TGF-β											
High	6–12	7	44	17	<0.17	76	15	0.34	44	17	0.19
Low	0–5	3	100	0		100	0		100	0	
NF-κB											
High	12	5	0	0	<0.0018^a^	53	25	0.11	0	0	0.0018^a^
Low	0–11	5	100	0		100	0		80	18	
MMP-7											
High	6–12	9	44	17	<0.90	76	15	<0.66	44	17	<0.92
Low	0–5	1	100	0		100	0	100	0		
COX-2											
High	6–12	7	43	19	0.38	69	19	0.93	43	19	0.88
Low	0–5	3	67	27		100	0		67	27	
HIF-1α											
High	6–12	7	29	17	<0.19	69	19	0.74	29	17	0.19
Low	0–5	3	100	0		100	0		67	27	

a P<0.05. IHC, immunohistochemistry; IRS, immunoreactive score; OS, overall survival; LC, local control; DFS, disease-free survival; SD, standard deviation. EGFR, epidermal growth factor receptor; MIB-1, molecular immunology borstel-1; HER2, human epidermal growth factor receptor type 2; ER, estrogen receptor; TNF-α, tumor necrosis factor-α; TGF-β, transforming growth factor-β; NF-κB, nuclear factor-κB; MMP-7, matrix metalloproteinase-7; COX-2; cyclooxygenase-2; HIF-1α, hypoxia-inducible factor-1α.
